# l-Tyrosine isopropyl ester

**DOI:** 10.1107/S1600536812042377

**Published:** 2012-10-20

**Authors:** Nelson Nuñez-Dallos, Klaus Wurst, Rodolfo Quevedo

**Affiliations:** a Universidad Nacional de Colombia, Sede Bogotá, Facultad de Ciencias, Departamento de Química, Cra 30 No. 45-03, Bogotá, 4-72 Colombia; b Institute of General, Inorganic and Theoretical Chemistry, University of Innsbruck, Innrain 80-82, 6020 Innsbruck, Austria

## Abstract

The title compound, C_12_H_17_NO_3_, adopts a folded conformation with a C—C(NH_2_)—C(=O)—O torsion angle of −95.9 (2)°. In the crystal, mol­ecules are linked by an O—H⋯N hydrogen bond, forming helical chains along the *b-*axis direction. Weak N—H⋯O and C—H⋯O hydrogen bonds are observed between the chains.

## Related literature

For information about tyrosine alkyl esters as prodrugs and the structure and inter­molecular inter­actions of l-tyrosine methyl ester compared to l-tyrosine and its ethyl and *n*-butyl esters, see: Nicolaï *et al.* (2011 ▶). For the *n*-butyl analogue, see: Qian *et al.* (2006 ▶). For macrocyclization of tyrosine alkyl esters with formaldehyde, see: Quevedo & Moreno-Murillo (2009 ▶); Nuñez-Dallos *et al.* (2012 ▶). For a related structure of tyramine, see: Quevedo *et al.* (2012 ▶).
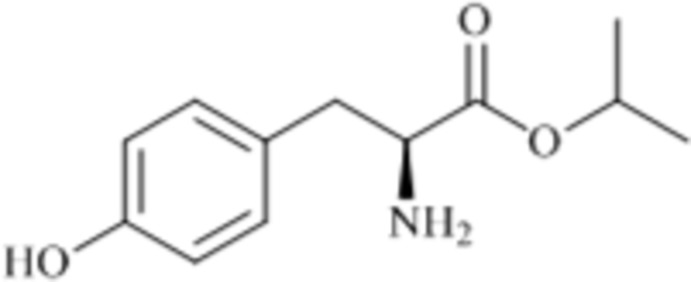


## Experimental

### 

#### Crystal data

C_12_H_17_NO_3_
*M* *_r_* = 223.27
Orthorhombic, 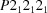

*a* = 5.4539 (1) Å
*b* = 14.0521 (3) Å
*c* = 16.5163 (4) Å
*V* = 1265.79 (5) Å^3^
*Z* = 4
Mo *K*α radiation
μ = 0.08 mm^−1^
*T* = 233 K
0.4 × 0.3 × 0.2 mm

#### Data collection

Nonius KappaCCD diffractometer
8375 measured reflections
1318 independent reflections
1271 reflections with *I* > 2σ(*I*)
*R*_int_ = 0.020

#### Refinement

*R*[*F* ^2^ > 2σ(*F* ^2^)] = 0.036
*wR*(*F* ^2^) = 0.099
*S* = 1.07
1318 reflections
158 parameters
H atoms treated by a mixture of independent and constrained refinement
Δρ_max_ = 0.17 e Å^−3^
Δρ_min_ = −0.15 e Å^−3^

### 

Data collection: *COLLECT* (Nonius, 1998 ▶); cell refinement: *SCALEPACK* (Otwinowski & Minor, 1997 ▶); data reduction: *DENZO* (Otwinowski & Minor, 1997 ▶) and *SCALEPACK*; program(s) used to solve structure: *SHELXS97* (Sheldrick, 2008 ▶); program(s) used to refine structure: *SHELXL97* (Sheldrick, 2008 ▶); molecular graphics: *Mercury* (Macrae *et al.*, 2008 ▶); software used to prepare material for publication: *SHELXL97*.

## Supplementary Material

Crystal structure: contains datablock(s) I, New_Global_Publ_Block. DOI: 10.1107/S1600536812042377/is5203sup1.cif

Structure factors: contains datablock(s) I. DOI: 10.1107/S1600536812042377/is5203Isup2.hkl

Supplementary material file. DOI: 10.1107/S1600536812042377/is5203Isup3.cml

Additional supplementary materials:  crystallographic information; 3D view; checkCIF report

## Figures and Tables

**Table 1:** Hydrogen-bond geometry (Å, °)

*D*—H⋯*A*	*D*—H	H⋯*A*	*D*⋯*A*	*D*—H⋯*A*
O3—H3*O*⋯N1^i^	0.97 (4)	1.78 (4)	2.736 (3)	167 (3)
N1—H1*N*⋯O3^ii^	0.88 (2)	2.27 (2)	3.106 (2)	157 (2)
N1—H2*N*⋯O3^iii^	0.89 (3)	2.46 (3)	3.336 (3)	171 (2)
C2—H2⋯O1^iv^	0.99	2.37	3.314 (3)	159

Symmetry codes: (i) 
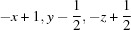
; (ii) 
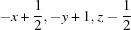
; (iii) 
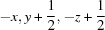
; (iv) 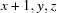.

**Fig. 1.**
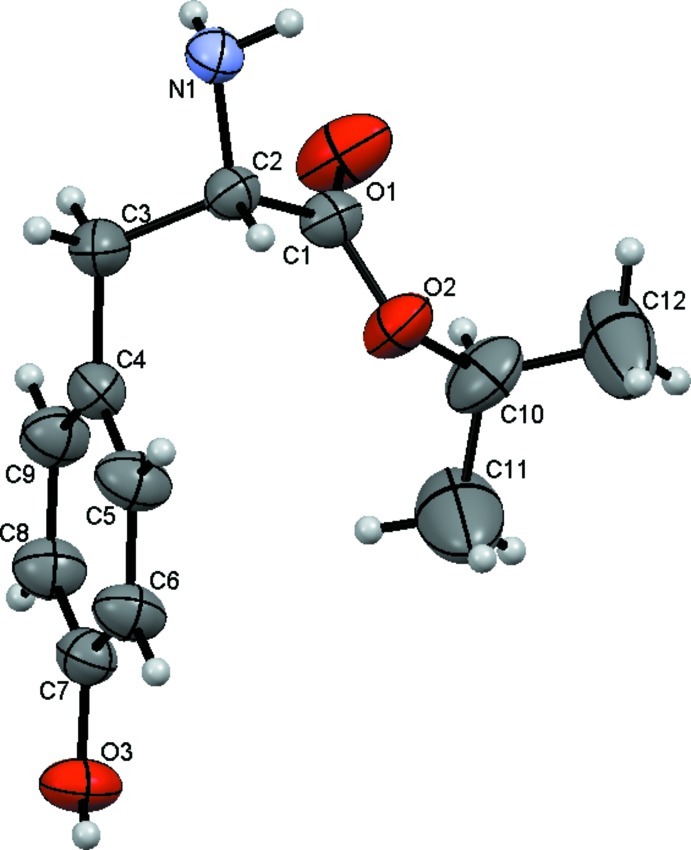
Molecular structure of the title compound with displacement ellipsoids drawn at the 50% probability level. Hydrogen atoms are shown as spheres of arbitrary radius.

**Fig. 2.**
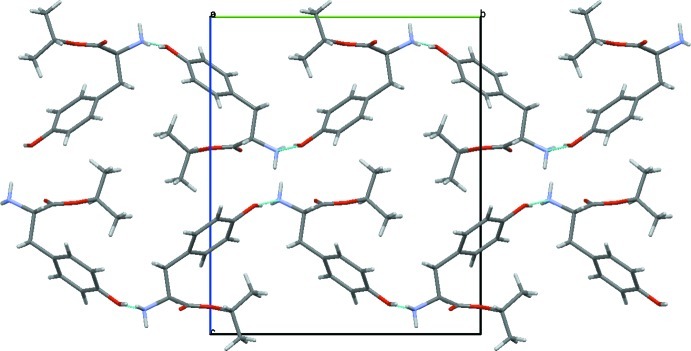
A view along the a axis of the crystal packing of the title compound. The O—H···N hydrogen bonds are shown as dashed cyan lines.

## supplementary crystallographic information

### Comment

*L*-Tyrosine alkyl esters are used as prodrugs for *L*-tyrosine due to these esters are more lipophilic and absorbed faster than *L*-tyrosine. In addition, they are hydrolyzed under physiological conditions (Nicolaï *et al.*, 2011). The crystal structures for a number of tyrosine esters have been determined: methyl, ethyl (Nicolaï *et al.*, 2011) and *n*-butyl esters (Qian *et al.*, 2006). Previous computational and spectroscopic studies of *L*-tyrosine isopropyl ester have suggested that the macrocyclization process with formaldehyde can be explained by the formation of a template in solution through intermolecular hydrogen bonds between the amino and the phenolic hydroxyl groups on adjacent molecules of *L*-tyrosine derivatives (Quevedo & Moreno-Murillo, 2009; Nuñez-Dallos *et al.*, 2012). We report here for the first time the crystal structure of *L*-tyrosine isopropyl ester.

The molecular structure of the title compound is shown in Fig. 1. The molecule adopts a folded conformation called U-shaped or scorpion conformation, as evidenced in the C1—C2—C3—C4 torsion angle of 58.2 (3)°. Despite the adoption of this conformation, there is no evidence for significant intramolecular C—H···π interactions. In terms of overall conformation, the structure of the title compound resembles that of the *n*-butyl (Qian *et al.*, 2006) and ethyl analogues (Nicolaï *et al.*, 2011). The crystal packing is stabilized by strong hydrogen bonds between the hydroxyl of the phenol group and the N-atom of the amine group (Fig. 2). Furthermore, molecules are connected into a three-dimensional array *via* N1—H1N···O3, N1—H2N···O3 and C2—H2···O1 intermolecular hydrogen-bonding interactions; see Table 1 for geometric parameters and symmetry operations.

### Experimental

Concentrated sulfuric acid (8 ml) was added to a suspension of *L*-tyrosine (10.00 g, 55.19 mmol) in isopropyl alcohol (40 ml). The mixture was heated at reflux and allowed to stir for 24 h. Then the reaction mixture was cooled to room temperature and placed into ice-cold water. The pH was brought to ~7 with concentrated ammonia, and isopropyl alcohol (40 ml) was added later to the reaction mixture. Precipitated ammonium sulfate was filtered off and washed with isopropyl alcohol (3×10 ml). The filtrate was concentrated under reduced pressure to a volume of 30 ml and single crystals were obtained by slow evaporation at room temperature. The title compound formed colorless prisms (5.50 g, 45%). m.p. 121–122 °C.

^1^H NMR (400 MHz, CDCl_3_) δ 7.03 (d, *J* = 8.4 Hz, 2H), 6.69 (d, *J* = 8.4 Hz, 2H), 5.03 (hept, *J* = 6.3 Hz, 1H), 3.65 (dd, *J* = 7.7, 5.4 Hz, 1H), 3.01 (dd, *J* = 13.7, 5.3 Hz, 1H), 2.79 (dd, *J* = 13.8, 7.7 Hz, 1H), 1.25 (d, *J* = 6.2 Hz, 3H), 1.22 (d, *J* = 6.3 Hz, 3H). ^13^C NMR (100 MHz, CD_3_OD) δ 21.9, 22.0, 41.1, 57.0, 69.7, 116.3, 128.9, 131.4, 157.4, 175.6. HRMS (ESI), *m/z* calcd for [C_12_H_17_NO_3_+H]^+^ 224.1281; found: 224.1279 [*M*+H]^+^, 246.1094 [*M*+Na]^+^, 222.1088 [M—H]^-^.

### Refinement

The H atoms on N1 and O3 were located in a difference map and refined isotropically [refined distances: N—H = 0.88 (2) and 0.89 (3) Å, and O—H = 0.97 (4) Å]. All H atoms bound to C atoms were refined using a riding model, with C—H = 0.94–0.98 Å and *U*_iso_(H) = 1.2 or 1.5 times *U*_eq_(C). In the absence of significant anomalous scattering effects, Friedel pairs have been merged in the final refinement.

### Crystal data

**Table:**

C_12_H_17_NO_3_	*F*(000) = 480
*M**_r_* = 223.27	*D*_x_ = 1.172 Mg m^−^^3^
Orthorhombic, *P*2_1_2_1_2_1_	Mo *K*α radiation, λ = 0.71073 Å
Hall symbol: P 2ac 2ab	Cell parameters from 13069 reflections
*a* = 5.4539 (1) Å	θ = 1.0–25.0°
*b* = 14.0521 (3) Å	µ = 0.08 mm^−^^1^
*c* = 16.5163 (4) Å	*T* = 233 K
*V* = 1265.79 (5) Å^3^	Prism, colorless
*Z* = 4	0.4 × 0.3 × 0.2 mm

### Data collection

**Table:**

Nonius KappaCCD diffractometer	1271 reflections with *I* > 2σ(*I*)
Radiation source: fine-focus sealed tube	*R*_int_ = 0.020
Graphite monochromator	θ_max_ = 25.0°, θ_min_ = 2.5°
Detector resolution: 9.1 pixels mm^-1^	*h* = −6→6
φ and ω scans	*k* = −16→16
8375 measured reflections	*l* = −19→19
1318 independent reflections	

### Refinement

**Table:**

Refinement on *F*^2^	Secondary atom site location: difference Fourier map
Least-squares matrix: full	Hydrogen site location: inferred from neighbouring sites
*R*[*F*^2^ > 2σ(*F*^2^)] = 0.036	H atoms treated by a mixture of independent and constrained refinement
*wR*(*F*^2^) = 0.099	*w* = 1/[σ^2^(*F*_o_^2^) + (0.054*P*)^2^ + 0.2704*P*] where *P* = (*F*_o_^2^ + 2*F*_c_^2^)/3
*S* = 1.07	(Δ/σ)_max_ < 0.001
1318 reflections	Δρ_max_ = 0.17 e Å^−^^3^
158 parameters	Δρ_min_ = −0.15 e Å^−^^3^
0 restraints	Extinction correction: *SHELXL*, Fc^*^=kFc[1+0.001xFc^2^λ^3^/sin(2θ)]^-1/4^
Primary atom site location: structure-invariant direct methods	Extinction coefficient: 0.059 (14)

### Special details

**Table:**

Geometry. All e.s.d.'s (except the e.s.d. in the dihedral angle between two l.s. planes) are estimated using the full covariance matrix. The cell e.s.d.'s are taken into account individually in the estimation of e.s.d.'s in distances, angles and torsion angles; correlations between e.s.d.'s in cell parameters are only used when they are defined by crystal symmetry. An approximate (isotropic) treatment of cell e.s.d.'s is used for estimating e.s.d.'s involving l.s. planes.
Refinement. Refinement of *F*^2^ against ALL reflections. The weighted *R*-factor *wR* and goodness of fit *S* are based on *F*^2^, conventional *R*-factors *R* are based on *F*, with *F* set to zero for negative *F*^2^. The threshold expression of *F*^2^ > σ(*F*^2^) is used only for calculating *R*-factors(gt) *etc*. and is not relevant to the choice of reflections for refinement. *R*-factors based on *F*^2^ are statistically about twice as large as those based on *F*, and *R*- factors based on ALL data will be even larger.

### Fractional atomic coordinates and isotropic or equivalent isotropic displacement parameters (Å^2^)

**Table:**

	*x*	*y*	*z*	*U*_iso_*/*U*_eq_	
N1	0.2421 (4)	0.74601 (12)	0.07769 (10)	0.0395 (4)	
H1N	0.241 (4)	0.7383 (17)	0.0246 (14)	0.049 (6)*	
H2N	0.096 (6)	0.7704 (18)	0.0881 (16)	0.056 (8)*	
O1	−0.1561 (3)	0.61052 (12)	0.07928 (14)	0.0699 (6)	
H3O	0.483 (7)	0.311 (2)	0.404 (2)	0.089 (10)*	
O2	0.1284 (3)	0.49667 (10)	0.08581 (10)	0.0539 (5)	
O3	0.3192 (3)	0.33815 (11)	0.40495 (10)	0.0528 (5)	
C1	0.0508 (4)	0.58592 (14)	0.09087 (12)	0.0392 (5)	
C2	0.2565 (4)	0.65246 (13)	0.11620 (11)	0.0364 (5)	
H2	0.4156	0.6228	0.1023	0.044*	
C3	0.2457 (5)	0.66834 (15)	0.20858 (12)	0.0495 (6)	
H3A	0.3792	0.7114	0.2239	0.059*	
H3B	0.0910	0.7004	0.2216	0.059*	
C4	0.2644 (4)	0.57967 (14)	0.25969 (11)	0.0418 (5)	
C5	0.4658 (5)	0.52015 (17)	0.25389 (15)	0.0501 (6)	
H5	0.5912	0.5351	0.2170	0.060*	
C6	0.4873 (4)	0.43895 (16)	0.30120 (14)	0.0469 (6)	
H6	0.6246	0.3990	0.2955	0.056*	
C7	0.3068 (4)	0.41695 (14)	0.35669 (12)	0.0401 (5)	
C8	0.1058 (4)	0.47529 (16)	0.36372 (14)	0.0479 (6)	
H8	−0.0183	0.4607	0.4012	0.057*	
C9	0.0861 (4)	0.55580 (16)	0.31541 (14)	0.0475 (6)	
H9	−0.0524	0.5951	0.3208	0.057*	
C10	−0.0553 (6)	0.42135 (18)	0.07362 (19)	0.0698 (8)	
H10	−0.2208	0.4471	0.0849	0.084*	
C11	0.0012 (13)	0.3444 (2)	0.1322 (2)	0.144 (2)	
H11A	0.0004	0.3701	0.1867	0.216*	
H11B	0.1617	0.3181	0.1203	0.216*	
H11C	−0.1215	0.2947	0.1278	0.216*	
C12	−0.0438 (11)	0.3886 (3)	−0.0106 (2)	0.135 (2)	
H12A	−0.0876	0.4405	−0.0465	0.203*	
H12B	−0.1574	0.3362	−0.0182	0.203*	
H12C	0.1214	0.3673	−0.0228	0.203*	

### Atomic displacement parameters (Å^2^)

**Table:**

	*U*^11^	*U*^22^	*U*^33^	*U*^12^	*U*^13^	*U*^23^
N1	0.0479 (11)	0.0363 (9)	0.0344 (9)	−0.0041 (8)	−0.0001 (8)	0.0030 (7)
O1	0.0357 (9)	0.0493 (9)	0.1246 (16)	−0.0022 (8)	0.0005 (11)	0.0016 (10)
O2	0.0557 (9)	0.0332 (7)	0.0729 (11)	0.0001 (7)	−0.0022 (9)	−0.0069 (7)
O3	0.0521 (10)	0.0499 (9)	0.0564 (9)	0.0071 (8)	0.0075 (8)	0.0213 (7)
C1	0.0385 (11)	0.0358 (10)	0.0433 (10)	0.0010 (9)	0.0073 (9)	0.0020 (9)
C2	0.0382 (10)	0.0325 (9)	0.0385 (9)	0.0019 (9)	0.0011 (9)	−0.0007 (7)
C3	0.0715 (15)	0.0377 (10)	0.0394 (10)	0.0042 (12)	−0.0017 (11)	0.0012 (8)
C4	0.0503 (12)	0.0400 (10)	0.0351 (9)	−0.0007 (10)	−0.0045 (9)	0.0012 (8)
C5	0.0470 (12)	0.0575 (13)	0.0457 (11)	0.0040 (11)	0.0071 (10)	0.0144 (11)
C6	0.0420 (12)	0.0513 (12)	0.0474 (11)	0.0083 (11)	0.0019 (10)	0.0103 (10)
C7	0.0418 (11)	0.0403 (10)	0.0382 (10)	−0.0027 (10)	−0.0033 (9)	0.0051 (9)
C8	0.0439 (12)	0.0503 (12)	0.0495 (12)	0.0010 (11)	0.0075 (10)	0.0089 (10)
C9	0.0445 (12)	0.0483 (12)	0.0498 (12)	0.0079 (10)	0.0025 (10)	0.0029 (10)
C10	0.0762 (19)	0.0401 (12)	0.093 (2)	−0.0166 (13)	0.0027 (17)	−0.0061 (13)
C11	0.268 (7)	0.081 (2)	0.082 (2)	−0.073 (4)	−0.018 (4)	0.0200 (19)
C12	0.230 (6)	0.109 (3)	0.0674 (19)	−0.105 (4)	−0.022 (3)	0.0063 (19)

### Geometric parameters (Å, º)

**Table:**

N1—C2	1.462 (2)	C5—H5	0.9400
N1—H1N	0.88 (2)	C6—C7	1.380 (3)
N1—H2N	0.89 (3)	C6—H6	0.9400
O1—C1	1.196 (3)	C7—C8	1.374 (3)
O2—C1	1.326 (2)	C8—C9	1.388 (3)
O2—C10	1.471 (3)	C8—H8	0.9400
O3—C7	1.366 (2)	C9—H9	0.9400
O3—H3O	0.97 (4)	C10—C12	1.467 (5)
C1—C2	1.519 (3)	C10—C11	1.483 (5)
C2—C3	1.543 (3)	C10—H10	0.9900
C2—H2	0.9900	C11—H11A	0.9700
C3—C4	1.508 (3)	C11—H11B	0.9700
C3—H3A	0.9800	C11—H11C	0.9700
C3—H3B	0.9800	C12—H12A	0.9700
C4—C9	1.380 (3)	C12—H12B	0.9700
C4—C5	1.384 (3)	C12—H12C	0.9700
C5—C6	1.388 (3)		
			
C2—N1—H1N	108.7 (15)	C5—C6—H6	120.1
C2—N1—H2N	108.1 (17)	O3—C7—C8	118.29 (18)
H1N—N1—H2N	103 (2)	O3—C7—C6	122.25 (19)
C1—O2—C10	118.1 (2)	C8—C7—C6	119.46 (18)
C7—O3—H3O	111 (2)	C7—C8—C9	120.0 (2)
O1—C1—O2	124.4 (2)	C7—C8—H8	120.0
O1—C1—C2	124.3 (2)	C9—C8—H8	120.0
O2—C1—C2	111.33 (18)	C4—C9—C8	121.8 (2)
N1—C2—C1	113.20 (17)	C4—C9—H9	119.1
N1—C2—C3	107.33 (15)	C8—C9—H9	119.1
C1—C2—C3	109.46 (17)	C12—C10—O2	109.0 (3)
N1—C2—H2	108.9	C12—C10—C11	112.4 (3)
C1—C2—H2	108.9	O2—C10—C11	107.1 (3)
C3—C2—H2	108.9	C12—C10—H10	109.4
C4—C3—C2	115.55 (17)	O2—C10—H10	109.4
C4—C3—H3A	108.4	C11—C10—H10	109.4
C2—C3—H3A	108.4	C10—C11—H11A	109.5
C4—C3—H3B	108.4	C10—C11—H11B	109.5
C2—C3—H3B	108.4	H11A—C11—H11B	109.5
H3A—C3—H3B	107.5	C10—C11—H11C	109.5
C9—C4—C5	117.29 (18)	H11A—C11—H11C	109.5
C9—C4—C3	121.7 (2)	H11B—C11—H11C	109.5
C5—C4—C3	121.0 (2)	C10—C12—H12A	109.5
C4—C5—C6	121.7 (2)	C10—C12—H12B	109.5
C4—C5—H5	119.2	H12A—C12—H12B	109.5
C6—C5—H5	119.2	C10—C12—H12C	109.5
C7—C6—C5	119.8 (2)	H12A—C12—H12C	109.5
C7—C6—H6	120.1	H12B—C12—H12C	109.5
			
C10—O2—C1—O1	−6.8 (3)	C3—C4—C5—C6	179.2 (2)
C10—O2—C1—C2	171.22 (19)	C4—C5—C6—C7	−1.1 (4)
O1—C1—C2—N1	−37.6 (3)	C5—C6—C7—O3	−179.8 (2)
O2—C1—C2—N1	144.43 (18)	C5—C6—C7—C8	0.8 (3)
O1—C1—C2—C3	82.1 (3)	O3—C7—C8—C9	−179.7 (2)
O2—C1—C2—C3	−95.9 (2)	C6—C7—C8—C9	−0.2 (3)
N1—C2—C3—C4	−178.6 (2)	C5—C4—C9—C8	−0.3 (3)
C1—C2—C3—C4	58.2 (3)	C3—C4—C9—C8	−178.6 (2)
C2—C3—C4—C9	−123.6 (2)	C7—C8—C9—C4	0.0 (3)
C2—C3—C4—C5	58.2 (3)	C1—O2—C10—C12	105.1 (3)
C9—C4—C5—C6	0.8 (3)	C1—O2—C10—C11	−133.1 (3)

### Hydrogen-bond geometry (Å, º)

**Table:**

*D*—H···*A*	*D*—H	H···*A*	*D*···*A*	*D*—H···*A*
O3—H3*O*···N1^i^	0.97 (4)	1.78 (4)	2.736 (3)	167 (3)
N1—H1*N*···O3^ii^	0.88 (2)	2.27 (2)	3.106 (2)	157 (2)
N1—H2*N*···O3^iii^	0.89 (3)	2.46 (3)	3.336 (3)	171 (2)
C2—H2···O1^iv^	0.99	2.37	3.314 (3)	159

Symmetry codes: (i) −*x*+1, *y*−1/2, −*z*+1/2; (ii) −*x*+1/2, −*y*+1, *z*−1/2; (iii) −*x*, *y*+1/2, −*z*+1/2; (iv) *x*+1, *y*, *z*.
